# Renal Protective Effect of Sirtuin 1

**DOI:** 10.1155/2014/843786

**Published:** 2014-10-16

**Authors:** Yi-jun Dong, Nian Liu, Zhi Xiao, Tao Sun, Shu-hui Wu, Wei-xia Sun, Zhong-gao Xu, Hang Yuan

**Affiliations:** ^1^Department of Nephrology, First Hospital of Jilin University, Changchun 130021, China; ^2^Department of Urology, First Hospital of Jilin University, Changchun 130021, China; ^3^Department of Nephrology, Second Hospital of Jilin University, Changchun 130021, China; ^4^Department of the Integrated Traditional Chinese and Western Medicine, Second Hospital of Jilin University, Changchun 130021, China

## Abstract

Silent information regulator 2 (Sir2) is a nicotinamide adenine dinucleotide- (NAD^+^-) dependent deacetylase. The homology of SIRT1 and Sir2 has been extensively studied. SIRT1 deacetylates target proteins using the coenzyme NAD^+^ and is therefore linked to cellular energy metabolism and the redox state through multiple signalling and survival pathways. During the past decade, investigators have reported that SIRT1 activity is essential in cancer, neurodegenerative diseases, diabetes, cardiovascular disease, and other age-related diseases. In the kidneys, SIRT1 may inhibit renal cell apoptosis, inflammation, and fibrosis. Therefore its activation may also become a new therapeutic target in the patients with chronic kidney disease including diabetic nephropathy. In this paper, we would like to review the protective functions of sirtuins and the role of SIRT1 in the onset of kidney disease based on previous studies, including diabetic nephropathy, acute renal injury, chronic kidney disease as well as lupus nephritis.

## 1. Introduction

Silent information regulator 2 (Sir2) is a nicotinamide adenine dinucleotide- (NAD^+^-) dependent deacetylase that was first discovered in the 1970s in yeast; Sir2 prolongs the lifespan of numerous species [1]. Mammals have seven different sirtuins (SIRT1–SIRT7), which share the same conserved catalytic core region composed of 275 amino acids. Each member has diverse physiological functions and different subcellular localisations [2]. The SIRT1 gene, composed of 500 amino acid residues, was first discovered in 1999. The homology of SIRT1 and Sir2 has been extensively studied. The 363rd histidine exhibits deacetylation activity. Aside from the acetylation of the histones H1 twenty-sixth, H3 ninth, and H4 sixteenth lysine, SIRT1 also deacetylates target proteins, such as protein 53, forkhead-box transcription factor 3, notch, and poly-ADP-ribose polymerases. Therefore, SIRT1 is linked to cellular energy metabolism and cell senescence regulation [2–5].

## 2. SIRT1 Overview

### 2.1. Discovery of SIRTs

In 1986, Ivy [6] isolated and identified a gene associated with the lifespan of cells from yeast. She also found the same gene in* Caenorhabditis elegans* and* Drosophila* sp. and named it Sir2. Scientists gradually identified a family of proteins that are homologous to Sir2 from mammalian cells and referred to them as sirtuins (SIRTs). In bacterial and mammalian evolution, SIRT genes are considerably conservative. Therefore, SIRT proteins possess high structural and functional homologies with Sir2. The first SIRT was discovered in the body of the SIRT protein and was named SIRT1. Since then, six SIRT proteins have been found (SIRT2 to SIRT7). SIRT1 and yeast Sir2, which have the highest homologies, have been extensively studied [7].

### 2.2. Structure and Distribution of SIRT1

SIRT1, a class III histone deacetylase, is highly conserved from bacteria to humans and is homologous to Sir2 in mammals. The human SIRT1 gene is located on chromosome 10q22.1. This gene comprises nine exons and eight introns and is approximately 33 kb long. The 5′- and 3′-ends of the gene have a nontranslated region that contains p53 and have 1793 bp with no splicing mutation. SIRT1 is mainly localised in the nucleus, but it can also be found in the cytoplasm, where it facilitates nuclear cytoplasmic shuttling. This gene is widely expressed in foetal and adult tissues, including fat and muscle tissues of the liver, kidneys, and brain. It is also uniformly expressed in islet cells but is rarely expressed in islet exocrine gland cells.

### 2.3. Biological Effects of SIRT1

SIRT1 has many substrates, such as histone p53, tumour suppressor transcription factor FOXO, AF618, peroxisome proliferator-activated receptor-coactivator-1 *α*, and KU70 [8]. SIRT1 mainly utilises deacetylase activity to exert its regulatory effects on various physiological processes, including gene transcription, energy metabolism, cell senescence, glucose metabolism, lipid metabolism, and insulin secretion [9] (Table 1).

## 3. Role of SIRT1 in the Kidney Disease

SIRT1 overexpression in renal inner medullary mesenchymal cells [10] indicates renal oxidative stress. SIRT1 is highly expressed in medullary tubular cells and moderately expressed in cortical proximal tubular cells. SIRT1 can protect and maintain kidney cell function. The effect of SIRT1 is negligible under normal conditions. As a humoural factor, SIRT1 can exert a powerful renal protective effect against ischaemic or toxic substance injury.

### 3.1. SIRT1 and Diabetic Nephropathy

Diabetic nephropathy (DN), a serious complication of diabetes, is a leading cause of mortality in patients with diabetes mellitus. Recent studies have shown that SIRT1 is closely related to the occurrence and development of DN. Maeda et al. [11] found that four single nucleotide polymorphisms (SNPs) in SIRT1 are associated with DN. Analysis of the haplotype of SIRT1 revealed that 11 sites are closely linked to DN in related SNPs. This finding shows that susceptibility to DN is closely related to SIRT1.

High glucose can lead to pathological changes in glomerular mesangial cells, podocytes, and filtration barrier [12]. High blood glucose levels are usually associated with increases in the expression of p38 and cleaved Caspase-3. The intermittent calorie restriction-induced increase in SIRT1 expression can significantly reverse the increase in the levels of the two substances; it can also block the increase in p53 content in the kidneys under hyperglycaemic conditions. Such a phenomenon can significantly mitigate the progression of rat DN [13]. Na^+^ and K^+^-ATPase are important indicators of kidney disease because they can reflect the damage level in the basement membrane. Yuan et al. [14] found that AMPK deactivation decreases SIRT1 expression, consequently reducing Na^+^ in rat renal proximal tubules and K^+^-ATPase activity. Another study [15] speculated that SIRT1 regulates the TGF-*β*/Smad and ERK1/2 pathways and inhibits basement membrane thickening in a diabetic mouse, thereby delaying the progression of early DN. Podocyte apoptosis usually occurs in rats glomeruli with DN. Chuang et al. [16] found that the accumulation of advanced glycation end products in diabetic patients promotes FOXO4 acetylation. This phenomenon initiates the transcription of proapoptotic protein Bcl-2, which mediates podocyte apoptosis. In another study, Chuang et al. using RNA interference-mediated SIRT1 knockdown mouse models observed serious albuminuria and mitochondrial dysfunction under diabetic condition, suggesting SIRT1 plays a key role in homeostatic maintenance of podocyte under mitochondrial stress/injury condition [17]. Moreover, Hasegawa et al. revealed that downregulation of SIRT1 and upregulation of the tight junction protein Claudin-1 by SIRT1-mediated epigenetic regulation in podocytes contributed to albuminuria under diabetic condition [18]. On the contrary, increased SIRT1 expression protects podocyte against apoptosis induced by the end products of sugar residues. In vivo experiments on db/db mice confirmed the results from the cell test. Kim et al. [19] found that the proapoptotic gene BAX is downregulated in db/db mice treated with resveratrol through gavage. Meanwhile, low phosphorylation levels of PI3K-Akt and FOXO3a as well as upregulation of the antiapoptotic gene Bcl-2 increase superoxide dismutases 1 and 2 (SOD1 and SOD2). Resveratrol can reverse the apoptosis of mesangial cells induced by glucose and oxidative stress in vitro. A study [20] found that NAD and resveratrol activate SIRT1, which can reverse the hyperglycaemia-induced mesangial cellular senescence. Conversely, SIRT1 inhibition by siRNA or nicotinamide can accelerate the senescence of mesangial cells. Kume et al. [21] found that SIRT1 blocks mesangial cell apoptosis induced by oxidative stress and TGF-*β*. Kitada et al. [22] detected a decrease in mitochondrial autophagy in the renal proximal tubule cells of diabetic rats subjected to calorie restriction. These results suggest that SIRT1 can prevent renal cell apoptosis under diabetic conditions and thus improve DN.

SIRT1 can also alleviate the inflammatory reaction in diabetic kidneys. Kitada et al. [22] found that Wistar diabetic rats have lower renal SIRT1 expression, higher urinary albumin excretion, lower creatinine clearance rate, and significantly higher contents of acetylated NF-*κ*B p65 and inflammatory factors than Wistar nondiabetic rats. Administration of SIRT1 agonists significantly reduces urinary albumin excretion, considerably improves creatinine clearance rate, and significantly decreases inflammatory factors. SIRT1 agonists substantially reverse WFR renal inflammation during factor upregulation and macrophage infiltration and then confer renal protection.

In addition, SIRT1 can alleviate diabetic renal fibrosis. Wu et al. [23] report that streptozotocin- (STZ-) induced diabetic rats were gavaged with resveratrol and detected that resveratrol increases SIRT1 and FOXO1 expression, decreases malondialdehyde and SOD activities, and reverses collagen IV and fibronectin synthesis, mesangial matrix accumulation, and glomerular tubular fibrosis. Matrix metalloproteinase-14 (MMP-14) was known as a target of SIRT1. Vasko et al. [24] reported that SIRT1 perpetrates nephrosclerosis through downregulation of MMP-14, which is relevant to fibrosis of vascular senescence. In another study, Shang et al. [25] injected 3,5-diiodo-l-thyronine (T2) into the peritoneum of STZ-induced diabetes rats. Compared with the diabetic rats, the experimental rats showed lower blood glucose, urine protein secretion, matrix expansion, TGF-*β*1, fibronectin, and collagen deposition after 12 weeks. However, the experimental rats had higher SIRT1 expression and activity than the control diabetic rats. In the in vitro study, mesangial cells were cultured in a high-glucose medium and showed a substantial decrease in fibronectin expression and collagen synthesis after T2 treatment. However, these in vitro and in vivo results showed renal protective effect of T2 is blocked by the SIRT1 inhibitor sirtinol. Therefore, T2 not only improves renal structure and function, but also restores SIRT1 expression in diabetic rats. These results suggest that T2 ameliorates DN by normalising SIRT1 expression.

**Figure 1:**
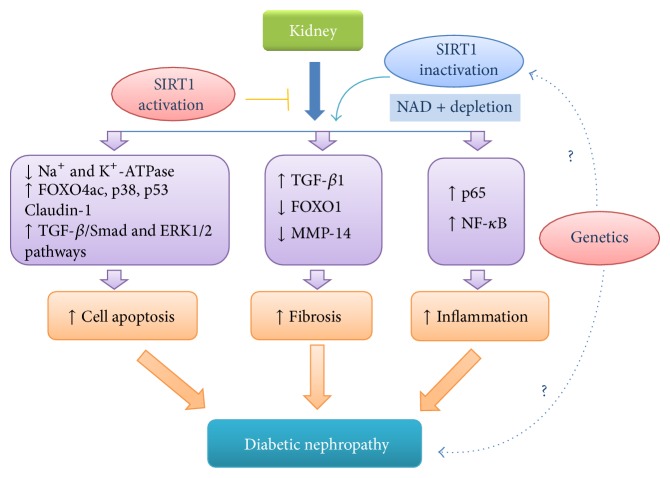
SIRT1 is closely related to the occurrence and development of diabetic nephropathy. Proposed pathogenesis of diabetic nephropathy (DN). NAD-dependent SIRT1 deacetylase may improve DN through the amelioration of these pathological changes. Genetic factors also may contribute to the activity of sirtuins.

In summary, SIRT1 alleviated DN by reducing renal cell apoptosis, relieving renal inflammation and fibrosis as shown in Figure 1.

### 3.2. SIRT1 and Acute Renal Injury

Acute renal failure decreases renal function (usually within 48 h). This condition confers significant harm to the patient. Therefore, the prevention and treatment of acute kidney injury (AKI) are particularly important. Studies have shown that SIRT1 activation can reduce various factors that induce AKI. Such factors include drugs, ischaemia-reperfusion, and endotoxins.

Cisplatin as a broad-spectrum antitumour drug is commonly used in clinical settings. The main side effect of cisplatin is AKI, with an incidence rate of 25% to 35%. Recent studies have shown that cisplatin-related AKI is caused by attenuated antioxidant effects [26]. Jung et al. [27] found that SIRT1 can attenuate the mediated nephrotoxicity of platinum compounds. Another study has shown that oxygen free radicals generated by cisplatin initiate oxidative damage in the mitochondria. This phenomenon causes mitochondrial vacuolation and increases the local production of oxygen free radical and the apoptosis of renal tubular cells, eventually resulting in AKI [28]. Resveratrol upregulates SIRT1 expression to alleviate the acute renal injury induced by cisplatin. SIRT1 exerts its effects through two mechanisms. In the first mechanism, SIRT1 upregulates catalase expression to degrade excessive free radicals, increase purine degradation, and promote ATP generation. This phenomenon leads to the inhibition of oxidative stress and the prevention of AKI. In the second mechanism, SIRT1 promotes PGC-1 transcription and increases the number and functions of mitochondria. These phenomena directly affect ATP synthesis for lipid metabolism and cell apoptosis, which are important in maintaining normal cell and organ functions. Mitochondrial dysfunction is an important pathological process for AKI caused by ischaemia or toxic substances [29]. Another study suggested that the short-term upregulation of SIRT1 in cisplatin-induced acute renal injury protects renal tubular cells against damage through core histone deacetylation or/and DNA repair by SIRT1 [30]. Kim et al. [31] cultured the proximal tubule cells of cisplatin-treated mice in vivo and confirmed that SIRT1 reduces p53 activity by reducing its acetylation level. SIRT1 also protects the cells against proximal tubular injury by inhibiting apoptosis.

Fan et al. [32] found that clamping in the renal artery of April-aged mice for 45 min can cause substantial kidney damage, as evidenced by increased serum creatinine and urea nitrogen levels and by the presence of tubular cell necrosis. However, February-aged mice only showed slight kidney injury. Other studies [33] have shown that SIRT1 expression is significantly higher in young mice than in adult mice. Pretreatment with the SIRT1 activator SRT-1720 can evidently improve the renal tubular injury caused by ischaemia-reperfusion in adult mice. Compared with wild-type mice, SIRT1 heterozygous mice exhibit more severe renal injury. In the SIRT^+/−^ gene UUO model group, the SIRT1 expression significantly decreased and was associated with cell apoptosis and interstitial fibrosis. However, administration of the SIRT1 agonist resveratrol inhibits cell apoptosis and fibrosis in wild-type UUO mice. Furthermore, SIRT1 reduces the level of acetylated p53, upregulates the expression of proliferating cell nuclear antigen, and inhibits the apoptosis of tubular cells by downregulating p21 expression. In the renal proximal tubule, SIRT1 expression can evidently improve the renal ischaemia-reperfusion induced by AKI [34].

AKI caused by endotoxaemia has received extensive attention. The kidney is the main metabolism and excretion route of toxins; lipopolysaccharides (LPSs) can be reabsorbed by the tubules. Hence, renal tubular epithelial cells possess high LPS concentration. High LPS concentration affects the structure and function of renal tubular epithelial cells and contributes to the occurrence and development of AKI [35]. Kalakeche et al. [36] showed that the S1 segment of the proximal tubule is the major uptaking region of endotoxins in vivo. However, S1 is not subjected to oxidative stress damage. The S2 segment, which is located downstream of the proximal tubule, exhibits serious structural and functional injuries. The SIRT1 in S1 cells is upregulated, whereas that in S2 cells is not changed. This result suggests that SIRT1 serves an important protective function against AKI. Hasegawa et al. [37] treated primary renal proximal tubule cells with 400 M TBHP for 6 h to mimic an acute oxidative situation. Damage in tubular cells promotes the phosphorylation and acetylation of multiple sites in the N and C terminals of intracellular p53, respectively. These phenomena activate p53 and thus induce apoptosis. However, SIRT1 can reduce its activity by reducing the acetylation level of p53, thereby cancelling its protective effect. Gao et al. [38] determined that SIRT1 exerts a protective effect against inflammatory kidney injury in endotoxaemia by suppressing the activation of STAT3, ERK1/2, and NF-*κ*B.

### 3.3. SIRT1 and Chronic Kidney Disease

The various body organs, particularly the kidney, suffer from different degrees of age-related damage. The kidney is vulnerable to specific age-related injuries. Therefore, the incidence of chronic kidney diseases develops along with age. Aging often leads to increased oxidative stress, free radical generation, and decreased antioxidant and free radical-scavenging activities. These findings suggest that oxidative stress is a significant cause of chronic kidney diseases. SIRT1 can protect cells from apoptosis induced by oxidative stress. Hao and Haase [39] observed that SIRT1 is overexpressed when renal medullary interstitial cells are exposed to high-permeability and low-oxygen environments. Downregulated SIRT1 expression significantly reduces oxidative stress resistance and triggers massive apoptosis. Conversely, activated SIRT1 promotes cell survival. This finding was verified in an in vitro unilateral ureteral obstruction model. SIRT1 directly or indirectly controls the activation of FOXO1, FOXO3, and FOXO4 through deacetylation and regulates cell response to oxidative stress [40]. By contrast, the aging individual is often accompanied by systemic hypoxia, which is closely related to apoptosis, metabolism, and cell cycle regulation. Renal senility is characterised by PI3K-Akt pathway downregulation. Studies have shown that PI3K and Akt activities decrease in the kidneys of aging rats under basal level or stress state [41]. Kume et al. [42] indicated that the body gradually suffers from hypoxia with aging. On the one hand, hypoxia activates the PI3K-Akt pathway, inhibits the expression of downstream FOXO3, and decreases SIRT1. On the other hand, hypoxia promotes FOXO3 acetylation, increases the expression of downstream target genes (p27Kip1 and Bnip3), and reduces transcription. Overall, hypoxia leads to the abnormal apoptosis and autophagy of senescent cells. Excessive oxidative stress leads to the accumulation of senescent cells. This accumulation leads to multiple cell injuries. In the kidney, TGF-*β*1 promotes the combination of HAT (p300) and Sp1 Smad and then upregulates p21, which is an important factor for glomerular hypertrophy [43]. Kume et al. [44] hypothesised that SIRT1 protects the kidney by directly interacting with Smad7 and inhibiting the p300-mediated acetylation of its lysine residues.

Adler et al. [45] suggested that NF-*κ*B can control senescence-specific gene expression and cell cycle through a conservative network system. NF-*κ*B is a complex compound composed of p50 and p65. NF-*κ*B activity can upregulate the expression of the COX-2 and TNF-*α* genes, which are important factors of inflammation and kidney senescence. The SIRT1 subunit interacts with p65, resulting in p53 deacetylation and then in NF-*κ*B transcription inhibition [46]. Liu et al. [47] found that increased acetylation of p65 and activator of transcription 3 (STAT3) mean increased binding with bromodomain and extraterminal (BET) proteins. Diabetic db/db mice with conditional deletion of SIRT1 in podocytes developed more proteinuria, kidney injury, and acetylation of p65 and STAT3 compared with db/db mice without SIRT1 deletion. These findings indicate that SIRT1 exerts a cytoprotective effect by alleviating cell apoptosis and inhibiting inflammatory reaction. Extracellular high-mobility group box 1 (HMGB1), release from stressed kidneys, acts as a potent proinflammatory cytokine that contributes to the pathogenesis of diverse inflammatory. Based on the deacetylase activity of SIRT1, Rabadi et al. [48] found SIRT1 participated in regulating nuclear retention of HMGB1 to ultimately modulate damage signaling initiated by HMGB1 secretion during stress and then eased increased renal damage.

### 3.4. SIRT1 and Lupus Nephritis (LN)

**Figure 2:**
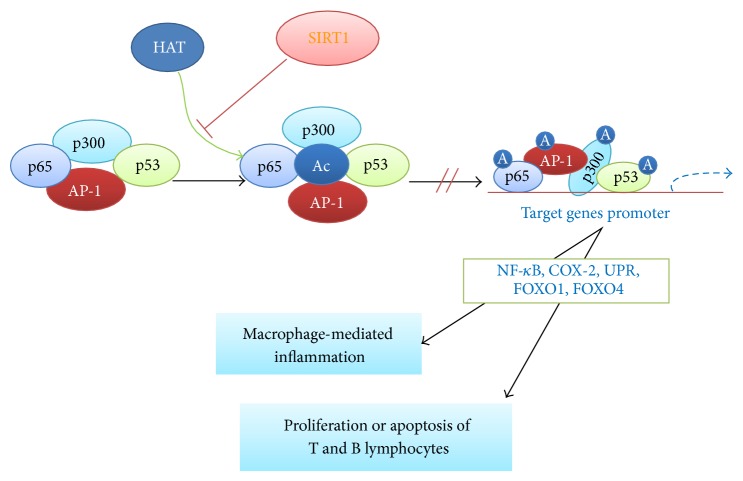
Role of SIRT1 in autoimmune disease. SIRT1 can regulate the proliferation or apoptosis of lymphocytes and macrophages that regulate the body's immune response.

Numerous studies have shown that SIRT1 activity is essential in cancer, neurodegenerative diseases, diabetes, cardiovascular disease, and other age-related diseases. LN is a common autoimmune disease, in which the production of autoimmune antibodies is the key process in its pathogenesis. SIRT1 knockout mice showed evident immunodeficiency. In addition, the IgM and IgG immune complexes were found deposited in the kidney and liver of these mice, showing lupus-like symptoms [49, 50]. Macrophages are the main immune cells of the innate immune system. They can produce a significant number of inflammatory cells, such as TNF-*α*, IL-6, and IL-1, during inflammatory response. SIRT1 can deacetylate p65 and AP-1 and then inhibit the NF-*κ*B pathway, AP-1 transcriptional activity, and COX-2 expression. SIRT1 can also reduce the proinflammatory phenotype of macrophages by regulating macrophage-mediated inflammation [51, 52]. The unfolded protein response (UPR) is another important signalling pathway utilised by macrophages. SIRT1 can regulate XBP1 expression involved in UPR [53]. In vivo study demonstrated that specific knockout of SIRT1 in bone marrow cells (MAC-SIRT1 KO) promotes the acetylation of p65 and renders NF-*κ*B hyperacetylated, resulting in increased transcriptional activation of proinflammatory target genes. Consistent with increased proinflammatory gene expression, Mac-SIRT1 KO mice challenged with a high-fat diet display high levels of activated macrophages in liver and adipose tissue, thus indicating that SIRT1 plays a pivotal role in regulating the inflammatory, immune, and apoptotic responses [54]. SIRT1 affects the activation, proliferation, and apoptosis of T and B lymphocytes by regulating FOXO1, FOXO3, and p53. SIRT1 knockout promotes the proliferation of mouse T cells; this phenomenon reduces the inhibition of NF-*κ*B and AP-1 transcription, which can be activated without the different CD28-stimulated secretions of effector T cells [49]. Taken together, these findings indicate that SIRT1 regulates the proliferation or apoptosis of numerous cells, thereby affecting the activities of lymphocytes and macrophages that regulate the body's immune response (Figure 2).

## 4. Conclusion

Taken together, SIRT1 depends on NAD^+^ through the acetylation or phosphorylation of different substrates. It exerts regulatory effects on gene silencing; cell proliferation, apoptosis, and senescence; glucose metabolism; and lipid homeostasis. SIRT1 is involved in the development of kidney diseases. SIRT1 can inhibit the apoptosis induced by kidney cell injuries, reduce renal inflammation, improve mitochondrial function, and reduce oxidative stress. Therefore, SIRT1 can improve DN and protect the kidney from acute injury, thereby delaying senescence and improving the prognosis of chronic kidney disease.

Considering the previously reported role of SIRT1 in kidney disease, it may become a new therapeutic target of kidney disease including DN. As a result, investigators have carried out studies based on caloric restriction and some SIRT1 activators, such as resveratrol or small molecule activator. The results are inspiring: SIRT1 does exert renoprotective effects by conferring resistance to cellular stresses such as hypoxia, reducing interstitial fibrosis, inhibiting tubular and glomerular cell apoptosis and inflammation. Conversely, SIRT1 inhibition mediated by siRNA and nicotinamide further confirmed the above results. However, despite a lot of experimental studies including their promising results and since there is presently very little evidence of pharmacological activity in humans, the number of clinical trials and their highlighting results are yet very limited. Due to a research of Spiegelman [55] as he mentioned, the pancreas activation of SIRT1 can increase the pancreas insulin secretion, resulting in gluconeogenesis in the liver, which may treat diabetes adversely. Neugebauer et al. [56] identified that SIRT1 broad-spectrum inhibitors have potential to reduce its focusing on adequate levels and tissue/cell-specificity by test on cancer cells. Therefore, further investigations, especially ones focusing on adequate levels and tissue/cell-specificity of manipulation of these pathways and human clinical trials, are necessary in the future.

## Figures and Tables

**Table 1:** Biological effects of SIRTs.

Substrates	Biological effects
FOXO, p53	Regulating cell cycle
Histones H1/H3/H4, Ku70	Alleviating cell apoptosis
FOXO1, PGC-1*α*	Alleviating glucose metabolism, lipid metabolism, and insulin secretion
FOXO1	Alleviating antioxidant
NF-*κ*B	Alleviating inflammatory reaction

p53: protein 53; FOXO: forkhead-box transcription factor; PGC-1*α*: peroxisome proliferator-activated receptor-coactivator-1 *α*; NF-*κ*B: nuclear factor-*κ*B.
